# Correlations between nutritional indicators and cognitive function in patients with stable schizophrenia in a hospital setting

**DOI:** 10.1371/journal.pone.0312227

**Published:** 2024-11-04

**Authors:** Binyou Wang, Yong Zhou, Han Yu, Techeng Jiang, Kezhi Liu, Jianlin Pu, Yilin Wang

**Affiliations:** Department of Psychiatry, Zigong Mental Health Center, The Zigong Affiliated Hospital of Southwest Medical University, Zigong, Sichuan Province, China; Shuguang Hospital, CHINA

## Abstract

**Background and objectives:**

Cognitive impairment is a core feature of schizophrenia, and it is now clear that there is a link between nutritional indicators and cognitive functioning. This study aimed to investigate correlations between three nutritional indicators (prognostic nutritional index [PNI], geriatric nutritional risk index [GNRI], and controlling nutritional status score [CONUT]) and cognitive function in hospitalized patients with stable schizophrenia.

**Methods:**

A total of 235 patients who were hospitalized with stable schizophrenia were included. Patient demographic information was collected through self-reports or electronic medical records, and cognitive function was assessed using the Montreal Cognitive Assessment in China (MoCA-C). Information on serum albumin and total cholesterol levels, lymphocyte counts, and body mass index during the stable stage of schizophrenia was collected to calculate the PNI, GNRI, and CONUT scores, according to their respective calculation criteria. Covariate-adjusted linear regression model and ordered logistic regression model were constructed to determine the relationship between nutritional indicators and cognitive function.

**Results:**

Overall, 90.2% of the patients were under the age of 60 years, and males comprised 60% of all patients. The median scores for MoCA-C, PNI, GNRI, and CONUT in hospitalized patients with stable schizophrenia were 18 (12,23), 52.85 (50.25,55.90), 110.85 (105.80,116.21), and 3 (3,3), respectively. The results of the correlation analysis showed that only PNI was associated with MoCA-C scores (r = 0.15, P = 0.021). This relationship was further confirmed by covariate-adjusted linear regression modeling (β = 0.147, 95%CI:0.049–0.351, p = 0.01) and ordered logistic regression modeling (OR = 0.054, 95%CI:0.001–0.106, p = 0.046).

**Conclusions:**

The findings revealed a significant correlation between PNI scores and MoCA-C scores in hospitalized patients with stable schizophrenia.

## Introduction

Schizophrenia is a severe and persistent mental disorder that affects over 21 million individuals and their families worldwide, resulting in significant mental stress and economic burden [1]. The symptoms usually involve disordered thinking, emotion, volition, cognitive functioning, and behavior, including hallucinations, delusions, emotional apathy, diminished volition, distraction, and memory loss [2–4]. Notably, cognitive impairment is a fundamental feature of schizophrenia that significantly impacts the patient’s quality of life, as well as the functional outcome and overall burden of the disease [5].

Schizophrenia typically necessitates long-term treatment and management in a medical facility. However, due to the unclear pathogenic mechanisms and the lack of objective biomarkers [6], cognitive assessment of patients with schizophrenia is heavily reliant on psychiatric scales, such as the Montreal Cognitive Assessment (MoCA) [7]. Unfortunately, the application of these scales requires significant time, effort, and clinical expertise. Therefore, there is a need to identify a simpler and more efficient tool for the rapid evaluation of cognitive function in these patients which would not only simplify the diagnostic process but also enable more accurate and comprehensive care and treatment.

A nutritional index is a measure of the quality and balance of a person’s diet. There are several nutritional indices, such as the prognostic nutritional index (PNI), geriatric nutritional risk index (GNRI), and the controlling nutritional status score (CONUT), which assess dietary quality and balance using readily accessible measurements such as lymphocyte counts, cholesterol levels, and serum albumin levels, as well as height and weight. Due to the ease of obtaining this information and the wide range of applicability of the scales, these indicators are commonly used for the screening and prognostic assessment of a variety of diseases, such as diabetes mellitus, cancer, COVID-19 and cardiovascular disease [8–13]. At present, several studies have reported an association between these three nutritional indices and cognitive function [14–16], suggesting their potential use as indicators of cognitive function. However, there are no studies on the relationships between these nutritional indicators and cognitive function in hospitalized patients with stable schizophrenia.

The current study aimed to investigate the associations between the three nutritional indices and cognitive function in hospitalized patients with stable schizophrenia. By examining the association between nutritional status, as indicated by the nutritional indices, and cognitive function, we aimed to identify a reliable indicator for the evaluation of cognitive function in patients with schizophrenia. Additionally, this study sought to enhance understanding of the role of nutrition in the treatment of schizophrenia and provide guidance for the clinical management of patients with schizophrenia in terms of dietary management and cognitive rehabilitation.

## Methods

### Informed consent and ethical approval process

The present study was executed as a cross-sectional survey, in strict alignment with the tenets of the Declaration of Helsinki. The diagnosis of schizophrenia was made by two trained psychiatrists in terms of criteria of the International Classification of Diseases, Tenth Revision (ICD-10), guidelines. Patients with stable schizophrenia were defined as those in whom the disease had remained stable for more than one month and the maintenance of an unaltered medication regimen for a minimum duration of two months before the commencement of the study. To ascertain the participants’ ability to grant informed consent, a standardized protocol was employed. This involved a thorough and detailed examination of their comprehension of the study’s objectives, methodologies, possible risks, and anticipated benefits, as well as their ability to arrive at an informed decision and understand the consequences of their participation. Once participants had demonstrated a clear understanding of these elements, they provided their consent by signing a written consent form on paper. The consent form was crafted to be straightforward and accessible, employing terminology that was readily comprehensible to individuals with schizophrenia. Sufficient opportunity was afforded to participants to peruse the consent form, seek clarifications, and discuss the study with a trusted person, should they desire to do so. The entire consent process, inclusive of the capacity assessment for stable schizophrenia patients, underwent rigorous examination and received the stamp of approval from the Institutional Review Board of the Zigong Mental Health Center (IRB2023024).

### Participant characteristics

Inpatients with stable schizophrenia were recruited and enrolled between August 1, 2023, and August 31, 2023, at the Zigong Mental Health Center in China. The inclusion criteria for patients were: 1) 18 years of age and older; 2) a diagnosis of stable schizophrenia by psychiatrists; 3) willingness to participate in the study and the provision of informed consent. The exclusion criteria were: 1) patients with autoimmune disease or who were undergoing antitumor therapy; 2) not diagnosed with stable schizophrenia; 3) the presence of severely impaired liver and kidney function; 4) patients who had not signed the informed consent form; 5) inability to calculate any of the three nutritional indicators.

### Assessment of cognition function

The extensive MATRICS Consensus Cognitive Battery (MCCB) is a pivotal instrument for cognitive assessment in this disorder. The concordance between MoCA and MCCB scores has been demonstrated in previous studies [17]. Thus, the cognitive function of the patients were assessed by expert psychiatrists using the Chinese Montreal Cognitive Assessment (MoCA-C) [18]. The MoCA-C scale must be completed within 15 min, and has a maximum score of 30 points, with lower scores indicating poorer cognitive function.

### Assessment of nutritional scores

The prognostic nutritional index (PNI) [19] was calculated using lymphocyte counts and serum albumin levels. A lower PNI indicates a high risk of malnutrition [10]. The PNI was calculated using the following formula:

$$\text{PNI}=10\times \text{serum}\;\text{albumin}\;\left(\text{g}/\text{dL}\right)+0.005\times \text{total}\;\text{lymphocyte}\;\text{count}\;\left(\text{per}\;{\text{mm}}^{3}\right)$$

The geriatric nutritional risk index (GNRI) [20] was determined using the serum albumin level and BMI of the patient. A lower GNRI indicates a higher risk of malnutrition [21]. The GNRI was calculated using the following formula:

$$\text{GNRI}=14.89\times \text{serum}\;\text{albumin}\;\left(\text{g}/\text{dL}\right)+41.7\times \text{BMI}/(22\;\text{kg}/{\text{m}}^{2})$$

The Controlling Nutritional Status (CONUT) [22] score was calculated using three variables, namely, serum albumin, total cholesterol levels and the lymphocyte count (Table 1). The CONUT score ranges from 0 to 12 and patients are classified into four nutritional states: normal, light, moderate, and severe [23].

**Table 1:** Assessment of nutritional status using the CONUT score.

Parameter	Normal	Light	Moderate	Severe
Serum albumin, g/dL	≥3.5	3.00–3.49	2.50–2.99	<2.50
Albumin score	0	2	4	6
Total cholesterol, mg/dL	>180	140–180	100–139	<100
Cholesterol score	0	1	2	3
Lymphocyte count, count/mL	≥1600	1200–1599	800–1199	<800
Lymphocyte score	0	1	2	3
Total score	0–1	2–4	5–8	9–12

Note: CONUT = the Controlling Nutritional Status

### Covariates

The information on covariates was collected through self-reports or electronic medical records. The information included disease duration (<5, 5–10, or >10 years), duration of hospitalization (<6 or ≥6 months), number of siblings (≤1 or ≥2), number of children (0 or ≥1), family history of mental disorders, first episode, marital status (married or unmarried/divorced/widowed), educational level (illiterate, high school and below, or university and above), vision problems, hearing problems, smoking history, history of falls, drinking history, COVID-19 history, number of chronic diseases (0 or 1 or ≥2), antipsychotic medications (typical, atypical, or combined), age (<60 or ≥60), sex, height, and weight. In addition, body mass index (BMI) values were calculated based on the weight and height of the patients and were divided into three groups, namely <18.5, 18.5–24, and ≥24. However, since only eight individuals had a BMI of <18.5, this group was combined with the 18.5–24 group, resulting in two groups based on BMI, namely, the <24 group and the ≥24 group.

The Generalized Anxiety Disorder 7 (GAD-7) scale was used to assess the severity of anxiety symptoms and their impact on functioning over the previous two weeks, while the Patient Health Questionnaire-9 (PHQ-9) was used to evaluate the severity of depression. Scores below 5 on these scales indicated the absence of anxiety and depression, respectively.

### Statistical analyses

Data were analyzed using SPSS 25.0 (IBM Corp., Armonk, NY, USA), with a two-sided P-value <0.05 considered statistically significant. Categorical variables are reported as numbers with corresponding percentages, while non-normally distributed continuous variables including age, BMI, disease duration (years), duration of hospitalization (months), and GAD-7 and PHQ-9 scores, are presented as medians and 25th and 75th percentiles (P25, P75).

The characteristics of the enrolled patients were compared using rank-sum tests. Variables that were found to be significantly associated with the MoCA-C scores (P < 0.05) were used for further adjustment. Spearman’s correlation coefficients were used to evaluate associations between nutritional indicators and MoCA-C scores. A covariate-adjusted linear regression analysis was then employed to determine the relationships between nutritional indicators and MoCA-C scores.

In light of the pronounced association identified between the PNI and MoCA-C scores in the current study population, we categorized the MoCA-C scores into quartiles of equal distribution to ascertain the nuanced correlations with PNI. To meticulously evaluate the relationship between PNI and MoCA-C scores, we conducted an ordered logistic regression analysis, which is specifically tailored for examining ordinal outcome variables.

## Results

### Characteristics of the study population

The characteristics of the inpatients with stable schizophrenia are shown in Table 2. A total of 235 patients with stable schizophrenia participated in the study, of whom 60% (141/235) were male and 40% (94/235) were female. Overall, 90.2% of the participants were under the age of 60 years, 54.9% had a body mass index greater than 24, 74.9% had a disease duration of more than 10 years, 83.4% had been hospitalized for more than six months, 85% had more than two siblings, 51.5% had no children, 22.6% had a family history of psychiatric disorders, 3% had first-episode schizophrenia, 19.6% were married, 90.2% had an educational level of high school or below or were classified as illiterate, 13.2% had vision problems, 7.2% had hearing problems, 41.7% had a history of smoking, 25.5% had a history of alcohol consumption, 6% had a history of falls, 39.1% had a history of COVID-19, 32.3% had other chronic conditions, 28.1% had depression, 15.3% had anxiety, and 93.2% used typical or atypical anti-psychotic medications.

**Table 2:** Characteristics of inpatients with stable schizophrenia.

Variable	N(%)	MoCA-C scores, median (P25,P75)	P-value
Total	235(100)	18(12,23)	
**Age(years), median(P25,P75)**			0.005
<60, 49(39,54)	212(90.2)	18.5(12.25, 24)	
≥60, 65(61,68)	23(9.8)	13(7,18)	
**Sex**			0.001
Male	141(60)	20(14,24)	
Female	94(40)	16(9,21)	
**BMI(kg/m** ^**2**^ **), median(P25,P75)**			0.06
<24, 21.7(20.1,23.2)	106(45.1)	19.5(14,24)	
≥24, 27.3(25.5,29.3)	129(54.9)	17(11,23)	
**Disease duration (years),median(P25,P75)**			0.203
<5, 3(2.5,4)	13(5.5)	20(13.5,22.5)	
5–10, 9(6.75,10)	46(19.6)	20(14.75,23.25)	
>10, 22.5(17,30.75)	176(74.9)	17(11,23.75)	
**Hospitalized time(months),median(P25,P75)**			0.168
<6, 3(2,4)	39(16.6)	20(13,24)	
≥6, 32.5(16,60.75)	196(83.4)	18(12,23)	
**Number of siblings**			0.154
≤1	33(14)	20(15,24)	
≥2	200(85)	18(11,23)	
**Number of children**			0.112
0	121(51.5)	20(12,24)	
≥1	114(48.5)	17(11,22)	
**Family history of mental disorder**			0.944
No	181(77.0)	18(12,23.5)	
Yes	53(22.6)	19(11,23)	
**First episode**			0.637
No	228(97)	18(12,23)	
Yes	7(3)	21(14,23)	
**Marital status**			0.007
Married	46(19.6)	15(9.5,20)	
Unmarried/divorced/ widowed	189(80.4)	19(12,24)	
**Education**			<0.001
Illiterate	16(6.8)	7(4.25,13)	
High school and below	196(83.4)	18(12,23)	
University and above	22(9.4)	24(21,27)	
**Vision problems**			0.107
No	204(86.8)	17(11,23)	
Yes	31(13.2)	21(17,24)	
**Hearing problems**			0.007
No	218(92.8)	18(12,24)	
Yes	17(7.2)	13(7,18)	
**Smoking history**			0.049
No	137(58.3)	17(11,22)	
Yes	98(41.7)	19(14,24)	
**Drinking history**			0.566
No	175(74.5)	18(12,24)	
Yes	60(25.5)	17(12.25,22.75)	
**Falls history**			0.001
No	221(94)	18(12.5,23.5)	
Yes	14(6)	10.5(6.25,13.75)	
**COVID-19 history**			0.008
No	141(60)	19(13,24)	
Yes	92(39.1)	16.5(9,21)	
**PHQ-9 Scores, median(P25,P75)**			0.917
<5, 1(0,3)	169(71.9)	18(11,24)	
≥5, 7(6,9)	66(28.1)	18(13.75,23)	
**GAD-7 Scores, median(P25,P75)**			0.573
<5, 0(0,2)	199(84.7)	18(12,24)	
≥5, 6.5(5,7.75)	36(15.3)	17(11,22)	
**Number of chronic diseases**			0.044
0	159(67.7)	19(13,24)	
1	40(17)	16(9.25,20.75)	
≥2	36(15.3)	16(9.5,21)	
**Anti-psychotics**			0.488
Typical	5(2.1)	24(13,25.5)	
Atypical	214(91.1)	18(12,23)	
Combined	16(6.8)	21(9.5,24.75)	

The median (P25, P75) MoCA-C score for the participants was 18 (12,23). Patients were assigned to two groups according to age, namely, <60 years and ≥60 years. The median (P25, P75) numbers of patients in these two groups were 49 (39, 54) and 65 (61, 68), respectively. Patients were also divided into two groups based on BMI (kg/m^2^), namely, <24 and ≥24, with median (P25, P75) values of 21.7 (20.1, 23.2) and 27.3 (25.5, 29.3), respectively. Three groups were used to classify patients according to disease duration (years): 5 years, 5–10 years, and >10 years, and the median (P25, P75) values for these groups were 3 (2.5, 4), 9 (6.75, 10), and 22.5 (17, 30.75), respectively. Two groups were set up for hospitalization time (months), namely, <6 and ≥6 months, with medians (P25, P75) of 3 (2, 4) and 32.5 (16, 60.75), respectively. Patients were also divided into two groups according to their GAD-7 scores, namely, <5 and ≥5, with medians (P25, P75) of 0 (0, 2) and 6.5 (5, 7.75), respectively. Finally, the patients were also allocated to two groups based on PHQ-9 scores, <5 and ≥5, with medians (P25, P75) of 1 (0, 3) and 7 (6, 9), respectively. It was found that the MoCA-C scores differed significantly in terms of patient age (p = 0.005), sex (p = 0.001), marital status (p = 0.007), educational level (p<0.001), presence of hearing problems (p = 0.007), history of smoking (p = 0.049), history of falls (p = 0.001), history of COVID-19 (p = 0.008), and the number of chronic illnesses (p = 0.044).

### Correlations between nutritional indicators and MoCA-C scale scores

Table 3 shows the results of the correlation analysis of the three nutritional indicators with the MoCA-C scale scores. The medians (P25, P75) of the PNI, GNRI, and CONUT scores were 52.85(50.25,55.90), 110.85(105.80,116.21), and 3(3,3), respectively. The Spearman’s correlation coefficients between the PNI, GNRI, and CONUT scores and the MoCA-C scale scores were 0.15, -0.053, and -0.094 respectively. Only the PNI was found to be significantly associated with the MoCA-C scale scores (p = 0.021), while no significant associations were found for the GNRI (p = 0.481) and CONUT (p = 0.149) scores.

**Table 3:** Analysis of the correlation between nutritional indicators and MoCA-C scale scores.

Variable	Median (P25,P75)	Spearman’s correlation coefficient	P-value
PNI	52.85(50.25,55.90)	0.15	0.021
GNRI	110.85(105.80,116.21)	-0.053	0.418
CONUT	3(3,3)	-0.094	0.149

Note: PNI = the Prognostic Nutritional Index; GNRI = the geriatric nutritional risk index; CONUT = the Controlling Nutritional Status score; MoCA-C = Montreal Cognitive Assessment, Chinese version.

Table 4 displays the correlation between the PNI and MoCA-C scale scores in patients with stable schizophrenia. Higher PNI scores (β = 0.217, 95%CI:0.123–0.466, p = 0.001) were significantly associated with higher scores on the MoCA-C scale in patients with stable schizophrenia, as shown in univariate linear regression analysis (Model 1). After adjustment for age, sex, marital status, education, hearing problem, smoking history, falls history, COVID-19 history, and number of chronic diseases, strong significant associations were found between higher PNI values and increased MoCA-C scores (β = 0.147, 95%CI:0.049–0.351, p = 0.01).

**Table 4:** Correlations between PNI and MoCA-C scale scores in patients with stable schizophrenia.

Variable	Model 1		Model 2	
	P-value	β(95%CI)	P-value	β(95%CI)
PNI	0.001	0.217(0.123–0.466)	0.01	0.147(0.049–0.351)

Note: MoCA-C = Montreal Cognitive Assessment, Chinese version; PNI = Prognostic Nutritional Index; Model 1: unadjusted model; Model 2: adjusted for age, sex, marital status, education, hearing problem, smoking history, falls history, COVID-19 history, and number of chronic diseases.

To further investigate the correlations between PNI and MoCA-C scores in this study population, we divided the MoCA-C scores into four equally sized quartiles and used ordered logistic regression analysis to examine the association between PNI and MoCA-C scores. The results revealed that PNI values were significantly associated with MoCA-C scores (OR = 0.054, 95%CI:0.001–0.106, p = 0.046, Table 5).

**Table 5:** Ordered logistic regression analysis of PNI on MoCA-C scores in patients with schizophrenia.

Variable	Wald Chi-Square	P-value	OR	95% CI
PNI	3.968	0.046	0.054	0.001–0.106

Note: PNI = Prognostic Nutritional Index; MoCA-C = Montreal Cognitive Assessment—Chinese version; Ordered logistic regression model: adjusted for age, sex, marital status, education, hearing problems, smoking history, falls history, COVID-19 history, and number of chronic diseases.

## Discussion

This study marks the first exploration of the associations between three nutritional indices (PNI, GNRI and CONUT) and cognitive performance in hospitalized individuals with stable schizophrenia. The findings revealed that only the PNI value was significantly correlated with the cognitive function, while the GNRI and CONUT scores failed to exhibit similar associations. Moreover, the PNI score can be easily calculated from routine laboratory blood analyses. Hence, the PNI score can serve as a simple indicator for evaluating the cognitive function of hospitalized patients with schizophrenia. This not only offers a convenient tool for clinicians but also a rapid assessment of the patient’s cognitive level by non-clinicians, providing valuable information for the care and management of schizophrenic patients and enhancing their quality of life.

Cognitive impairment in schizophrenia is influenced by a variety of factors, including 1) the underlying pathophysiology of the disease, where psychiatric and neurological changes can potentially cause cognitive decline [24, 25]; 2) antipsychotic medications, which, while essential, may lead to cognitive dysfunction and metabolic side effects that exacerbate impairment [26]; 3) the disease duration, as cognitive function tend to worsen over time. Additionally, it is documented that there is a strong correlation between nutritional status and cognitive function [27, 28].

In China, patients with schizophrenia often receive inpatient care in psychiatric hospitals where they are exposed to monotonous diets, vitamin deficiencies, limited physical activity, and confined living environments that can heighten the risk of malnutrition, which in turn heightens the risk of cognitive decline. As such, assessing the nutritional status of patients with schizophrenia is crucial for understanding their cognitive health and for prompt intervention. Addressing malnutrition can improve their nutritional well-being and potentially facilitate cognitive recovery.

In this study, we utilized three straightforward nutritional indices, GNRI, PNI, and CONUT, derived from routine blood test results to evaluate the nutritional status of patients with schizophrenia. The GNRI is determined from serum albumin levels and the BMI, while the PNI score is derived from lymphocyte counts and albumin concentrations, and the CONUT score is calculated from serum albumin and cholesterol levels and the lymphocyte count. BMI serves as a surrogate marker for body fat content, while cholesterol fulfills diverse roles in the nervous system. Changes in lymphocyte numbers or functions often signal the presence of underlying inflammatory conditions, and albumin levels reflect the body’s protein stores and its capacity for protein synthesis. Research has indicated that lower BMI values may be indicative of malnutrition, which can compromise both physical and cognitive function [29]. Furthermore, previous research has linked cholesterol, lymphocyte, and albumin levels to cognitive function [30–32]. Given these associations, it is plausible that these three nutritional indices are related to cognitive function in different diseases.

Indeed, the correlations between the nutritional indices PNI, GNRI, and CONUT with cognitive function have been validated in a wide variety of populations and geographic regions [14, 15, 33]. However, the present study revealed specific associations among inpatients with stable schizophrenia, in which only high PNI levels were linked to a decreased risk of cognitive dysfunction, while GNRI and CONUT scores showed no such associations. This suggests that these nutritional indicators influence cognitive function differently in this specific population.

A previous study [34] examining the use of GNRI and PNI as predictors of falls in 542 hospitalized patients with schizophrenia (64.6% male, mean age 53.8 ± 9.7 years) found that the PNI score, but not the GNRI score, was significantly associated with falls. Given the well-documented link between cognitive decline and falls [35, 36], this suggests that cognitive impairment is more closely associated with PNI than with GNRI. Our findings are consistent with this pattern, highlighting the value and relevance of the PNI in the context of schizophrenia. Notably, the CONUT scale, in contrast to the PNI, includes total cholesterol as an additional indicator, and categorizes nutritional status into four distinct states using a scoring system that assigns different values based on index levels. However, this method of calculation may not adequately capture the complexity of the body’s nutritional status, which could explain the lack of correlation observed in our study. Based on the above results, it is suggested that the PNI score is a simple and suitable tool for evaluating the cognitive function of hospitalized patients with schizophrenia.

Moreover, we categorized cognitive functions into three categories based on previous research: severe cognitive deficits (MoCA-C scores below 25), mild cognitive deficits (MoCA-C scores ranging from 25 to 26), and normal cognitive function (MoCA-C scores of 27 or above) [17]. The result revealed that higher PNI values are significantly correlated with a reduced risk of mild cognitive deficits (S1 Table). But the small sample sizes of mild cognitive deficits (n = 20) and normal populations (n = 20) may lead to limitations in the applicability and broad representativeness of this findings.

There were potential limitations to this study. Firstly, the study was conducted in only one healthcare organization with a small sample size and may therefore not be generalizable to other populations. Secondly, as the study was observational, it may not have taken into account confounding factors such as differences in treatments and dietary structures. Third, this study was cross-sectional and thus cannot analyze the causal relationship between PNI and cognitive function. Fourthly, the scales used in the study were scored by three psychiatrists who despite professional training, could have introduced a certain amount of subjectivity into the scoring process.

To overcome these limitations, future studies should include prospective cohort studies to validate the observed associations, gather a more substantial number of individuals with mild cognitive impairment and normal cognition, and minimize the effects of confounding factor bias through accurate data collection and careful analysis. Furthermore, the inclusion of patient populations across geographical and cultural boundaries would make the results more applicable.

## Conclusions

The findings revealed a significant correlation between PNI scores and cognitive function in hospitalized patients with stable schizophrenia.

## Supporting information

S1 Table Correlations between PNI and cognitive function in patients with stable schizophrenia. (DOCX)

S1 Data (XLSX)
