# Supplementary material for: Correlations between nutritional indicators and cognitive function in patients with stable schizophrenia in a hospital setting
Source: PLoS One. 2024 Nov 4;19(11):e0312227. doi: 10.1371/journal.pone.0312227 (PMC11534214; doi:10.1371/journal.pone.0312227)
Supplement: S1 Table — (DOCX) [file pone.0312227.s001.docx]

**Correlations between nutritional indicators and cognitive function in patients with stable schizophrenia in a hospital setting**

**SUPPLEMENTARY INFORMATION**

Binyou Wang^1¶^, Yong Zhou^1¶^, Han Yu^1^, Techeng Jiang^1^, Kezhi Liu^1^, Jianlin Pu^1 *^, Yilin Wang^1 *^

^1^ Department of Psychiatry, Zigong Mental Health Center, the Zigong Affiliated Hospital of Southwest Medical University, Zigong, Sichuan Province, China.

*Corresponding author

E-mail: wangyilinzg0321@163.com (YLW); pujianlin8899@163.com (JLP)

^¶^These authors contributed equally to this work.

**Table S1. Correlations between PNI and cognitive function in patients with stable schizophrenia.**

| **Variable** | **Mild cognitive deficit, n=20** | | **Severe cognitive deficits, n=195** | |
| --- | --- | --- | --- | --- |
|  | **P-value** | **OR (95%CI)** | **P-value** | **OR (95%CI)** |
| PNI | 0.019 | 0.740(0.576-0.952) | 0.069 | 0.901(0.808-1.008) |

Note:

PNI = Prognostic Nutritional Index; severe cognitive deficits: Montreal Cognitive Assessment --Chinese version (MoCA-C) scores below 25; mild cognitive deficits: MoCA-C scores ranging from 25 to 26; normal cognitive function: MoCA-C scores of 27 or above; Logistic regression model: adjusted for age, sex, marital status, education, hearing problems, smoking history, falls history, COVID-19 history, and number of chronic diseases.
